# Some Characteristics of Free Cell Population in the Airways of Rats after Intratracheal Instillation of Copper-Containing Nano-Scale Particles

**DOI:** 10.3390/ijms151121538

**Published:** 2014-11-24

**Authors:** Larisa I. Privalova, Boris A. Katsnelson, Nadezhda V. Loginova, Vladimir B. Gurvich, Vladimir Y. Shur, Yakov B. Beikin, Marina P. Sutunkova, Ilzira A. Minigalieva, Ekaterina V. Shishkina, Svetlana V. Pichugova, Ludmila G. Tulakina, Svetlana V. Beljayeva

**Affiliations:** 1 The Medical Research Center for Prophylaxis and Health Protection in Industrial Workers, 30 Popov Str., Ekaterinburg 620014, Russia; E-Mails: privalovali@yahoo.com (L.I.P.); loginova.Nadzhda@mail.ru (N.V.L.); gurvich@ymrc.ru (V.B.G.); sutunkova@ymrc.ru (M.P.S.); ilzira-minigalieva@yandex.ru (I.A.M.); 2 The Institute of Natural Sciences, the Ural Federal University, Ekaterinburg 620000, Russia; E-Mails: vladimir.shur@usu.ru (V.Y.S.); ekaterina.shishkina@labfer.usu.ru (E.V.S.); 3 The City Clinical Diagnostics Centre, 28 Dekabristov Str., Ekaterinburg 620142, Russia; E-Mails: beikin@mail.ru (Y.B.B.); ekb-lem@mail.ru (S.V.P.); sdiklein@gmail.com (L.G.T.); kdc_boss@mail.ru (S.V.B)

**Keywords:** ultrafine copper-containing particles, bronchoalveolar lavage, cytotoxicity

## Abstract

We used stable water suspensions of copper oxide particles with mean diameter 20 nm and of particles containing copper oxide and element copper with mean diameter 340 nm to assess the pulmonary phagocytosis response of rats to a single intratracheal instillation of these suspensions using optical, transmission electron, and semi-contact atomic force microscopy and biochemical indices measured in the bronchoalveolar lavage fluid. Although both nano and submicron ultrafine particles were adversely bioactive, the former were found to be more toxic for lungs as compared with the latter while evoking more pronounced defense recruitment of alveolar macrophages and especially of neutrophil leukocytes and more active phagocytosis. Based on our results and literature data, we consider both copper solubilization and direct contact with cellular organelles (mainly, mitochondria) of persistent particles internalized by phagocytes as probable mechanisms of their cytotoxicity.

## 1. Introduction

**Figure 1:**
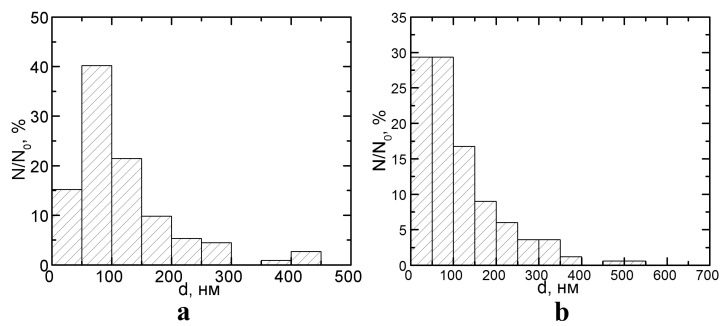
Percentage distributions of particles by size in the submicron range on filters through which the ambient air of a copper smelting and casting facility was drawn: (**a**) anode (crude) or (**b**) cathode (refined) copper (N—number of particles of a given diameter, N_0_—total number of particles).

Nanoparticles (NPs) of metals and of their oxides are of special interest for industrial toxicologists because, along with engineered metal-containing NPs, there exists usually a substantial fraction of nanoscale (“ultrafine”) particles of the same or chemically close substances within the particle size distribution of condensation aerosols generated by arc-welding and metallurgical technologies. In these aerosols, concomitant fractions are typically represented by micrometer particles (MPs) including submicron ones with dimensions >100 nm. In our experience, just such a situation is characteristic of workroom air pollution in copper smelters and copper refineries, as illustrated in Figure 1.

Which fraction of such aerosols (0–100 or >100–500 nm) is more noxious when deposited in the lungs? We put aside the probable difference of the deposition itself, which for both submicron fractions, being due to the diffusion mechanism, depends on particle size, irrespective of its density and, thus, its chemical nature. The latter, however, is of the highest importance as a determinant of a deposited particle’s biological toxicity, and this intrinsic bio-aggressivity (toxicity) should be assessed for specific NPs and MPs containing particular metals.

Although engineered copper and copper oxide NPs with Cu to O ratios in their composition were demonstrated to produce toxic effects in a number of published studies [1,2,3,4,5,6,7,8,9,10,11,12,13,14,15], the relevant literature is short of *in vivo* toxicity assessments on laboratory mammals. Recently we [16] demonstrated that 20 nm copper oxide particles, when injected intraperitoneally to rats at a dose of 10 mg/kg (0.5 mg per mL of deionized water) 3 times a week up to 19 injections, induced shifts in various functional and biochemical indices of the organism’s status as well as pathological changes in liver, spleen, kidneys and brain microscopic structure and augmented DNA fragmentation in cells of these organs as measured by the RAPD (Randomly Amplified Polymorphic DNA) test. The CuO nanoparticles used in that experiment, whilst being highly stable in a suspension on deionized water, were found to dissolve very quickly if normal saline or a biological fluid was added to it; moreover, some findings of our toxicological experiments were in agreement with the important role of Cu-ion release assumed by some authors as the main cause of the high cytotoxicity of copper oxide nanoparticles [2,4,5,6]*.*

In this connection, it should be noted that the cytotoxicity characteristic cited as a major harmful property of various metal-containing nanoparticles, including copper and copper oxide ones, is typically based on the findings of experiments *in vitro* carried out on cell cultures of various stable lines [1,4,5,6,12,13]*.* On the contrary, in our previous studies, the cytotoxicity of magnetite (Fe_3_O_4_) [17,18,19,20], silver and gold [21] NPs was assessed *in vivo* by changes in free cellular populations of deep airways after single intratracheal instillations of these materials. We studied cells of bronchoalveolar lavage fluid (BALF) obtained 24 h after intratracheal (i.t.) instillation to rats of small doses of nanoparticles (NPs) or of their micrometric counterparts (MPs) using optical (OM), transmission electron (TEM) and semi-contact atomic force (sc-AFM) microscopy. In this way, the i.t. model provides natural objects for studying the phagocytic activity of pulmonary (alveolar) macrophages and polymorphonuclear leukocytes, as well as intracellular localization of NPs engulfed by them and ultrastructural damage caused to the cell by internalized NPs.

The results thus obtained might be regarded as comparable with those obtained by other researchers in experiments on cell cultures, but we maintain that the former provided a valuable addition to *in vitro* assessments of particle cytotoxicity even if only because *in vivo* interaction between cells and particles occurs in a microenvironment which is not reproducible by any artificial culture medium.

Besides, quantitative characterization of BALF cells permits one to compare the ability of NPs and MPs to be recognized and dealt with by one of the most important physiological defense mechanisms against pulmonary deposition of any particles, namely the phagocytic response. The adequacy of this model for solving these problems is above any serious doubt as it has been shown repeatedly that the important qualitative and quantitative patterns of the response under consideration (in particular, its dependence on the cytotoxicity of deposited particles) observed in inhalation exposures to dusts are, in principle, the same in the case of their i.t. administration [22,23,24,25].

Specifically, it has become evident from our previous studies on iron oxide and silver particles [17,18,19,20,21] that both the recruitment of phagocytes toward the free surface of lower airways evoked by the deposition of metallic NPs and the phagocytic activity of these cells were more pronounced than similar responses to the deposition of even small (1 mcm) MPs, while damage to cells induced by NPs was more pronounced than that induced by such MPs. Our data also suggested that both the defense mechanism and the cytotoxic action depend on both NP dimensions and their chemical characteristics.

It was too early, however, to affirm that these dependences might be considered as a general nanotoxicological pattern. For the purpose of demonstrating to what extent they could be related to copper-containing NPs as well, we present in this article the data of a similar study involving specially prepared copper-containing ultrafine particles modeling various fractions of the above-mentioned industrial aerosols formed during copper smelting and casting.

## 2. Results and Discussion

### 2.1. Optical Microscopy Data

Table 1 presents the results of estimating shifts in a BALF cell population in response to intratracheal (i.t.) instillation of suspensions of copper-containing NPs and submicron MPs in comparison with the BALF of rats instilled with the same de-ionized water on which these suspensions were prepared (see Section 3). It can be seen that (1) both NPs and MPs cause a substantial increase over the control value in the number of neutrophil leukocytes (NL) as well as a significant but less pronounced increase in the number of alveolar macrophages (AM) in the BALF and, thus, a sharp increase in the NL/AM ratio; (2) this ratio is considerably and statistically significantly higher for NPs as compared with MPs due to both a much higher NL count and a somewhat lower AM count.

**Table 1:** Number of cells in the bronchoalveolar lavage fluid (BALF) 24 h after the intratracheal instillation of 1 mL suspension of copper-containing particles to rats at a dose of 0.5 mg per rat (*x* ± *S_x_*).

Particles Administered	Number of Cells * (×10^6^)			NL/AM
	Total	Neutrophil Leukocytes (NL)	Alveolar Macrophages (AM)	
Nanoparticles	12.42 ± 1.89 *^,^^●^	9.8 ± 2.16 *^,^^●^	2.44 ± 0.38 *	4.76 ± 1.39 *^,^^●^
Submicron particles	6.79 ± 1.28 *	3.64 ± 0.90 *	3.06 ± 0.86 *	1.39 ± 0.16 *
None (controls)	1.06 ± 0.14	0.052 ± 0.01	0.95 ± 0.18	0.06 ± 0.01

Statistically significant difference * from control group; ^●^ from submicron particles group (*p* < 0.05 by Student’s *t*-test).

Recruitment of phagocytizing cells into the lower airways, manifesting itself in an increased number of BALF cells, is a typical reaction to the deposition of particles in them. Both the total cell count and the shift towards polymorphonuclear (mainly neutrophil) leukocytes (NL) become the more marked, the stronger the damaging action of cytotoxic particles on alveolar macrophages (AM) [22,23,24,25]. The dependence of both indices on the number of destroyed AMs was experimentally modeled by: (1) intratracheal instillation of aseptically obtained peritoneal macrophages destroyed (without pre-incubation of these cells with any particles) by repeat freezing/thawing, or by ultrasonication; and (2) lipids extracted from these macrophage breakdown products. Such a dose-dependent imitation of the pattern of phagocyte recruitment toward cytotoxic particles with the help of macrophage breakdown products, on the one hand, and the good rank correlation (demonstrated in the same studies) between the above NL/AM shift and *in vitro* estimates (with the trypan blue exclusion test) of the capacity of different particulates to damage cultured peritoneal macrophage, on the other hand, justifies the usage of NL/AM ratio as a circumstantial, but rather informative comparative *in vivo* index for particle cytotoxicity*.*

It was also shown [22,23] that macrophage breakdown products less actively stimulated the recruitment of viable AMs compared with the recruitment of NLs. This may explain why under the impact of highly cytotoxic particles the resultant AM count is usually less increased over the control level as compared with the increase in the NL count, or is not increased at all, or sometimes is even decreased.

Thus the most obvious inference from the data presented in Table 1 is that the copper-containing NPs tested are much more cytotoxic than submicron MPs. It is more difficult, however, to attribute this difference definitely to the difference in particle diameter (20 and 340 nm, respectively) than it was in our studies comparing NPs of iron oxide [17] or silver [21] with chemically identical 1 μm MPs. As is shown in Section 3 below, copper-containing NPs and MPs are not strictly identical in their chemical composition: the former are composed entirely of copper oxide (nominally, CuO) while the latter have a metallic (elemental Cu) core and an 80 nm thick surface layer of another copper oxide (nominally, Cu_2_O). In our earlier studies of NPs of other metals we showed that the cytotoxicity of NPs of the same chemical nature increased with a decrease in their size, whereas that of NPs of the same size depended on their chemical nature. We believe that in the new case study discussed in this paper it would be justified to suggest that both factors had a part to play. A derivative of both factors is a significant difference in the solubility of NPs and MPs (see Section 3). Note also that a lot of researchers maintain that CuO nanoparticle intracellular solubilization with the release of Cu^2+^ ions plays the most important part as a mechanism of their cytotoxicity [2,4,5,6]*.*

As for the other aspect of the NL recruitment towards the free surface of the lower airways in response to the deposition of particles, nanoscale ones included, this recruitment is quite often described as “inflammation” and, thus, as a pathological phenomenon. However, there are fairly strong reasons for considering this response to be an important mechanism of partial compensation for the damage caused by cytotoxic particles to the alveolar macrophage, the main effector of pulmonary clearance [22,23,24]. Our experiments with Fe_3_O_4_ (magnetite) [17], gold and silver [21] nanoparticles confirm that NL recruitment and phagocytic activity played the same useful role as was evidenced, among other things, by a marked internalization of all NPs studied within both NLs and AMs. In the case of copper-containing NPs, similar evidence is presented and discussed below (sub-Section 2.3).

However, before proceeding to that part of this discussion, we propose discussing the shifts in some biochemical characteristics of the cell-free BALF, which also may testify to a higher bio-aggressivity of CuO-NPs as compared with submicron Cu_2_O/Cu MPs.

### 2.2. Some Indices of BALF Biochemistry

Cytotoxic damage to phagocytizing cells by particles is also indicated by shifts in some biochemical characteristics of BALF. Judging by the data in the scientific literature, the oft-used biochemical criterion of the comparative cytotoxicity of inhaled or instilled particles is the increased activity of lactate dehydrogenase (LDH) in the supernatant of BALF, which is usually explained by the release of this enzyme as a result of damage caused to the phagolysosomes of macrophages and of epithelial cells into the cytoplasm, and then on into the extracellular fluid, as according to the data of [26], for example. LDH concentration in the BALF 24 h after an instillation of highly cytotoxic DQ12 standard quartz particles was raised to a greater extent than after an instillation of TiO_2_ nanoparticles but to a much lower extent than after an instillation of Co and, especially, Ni nanoparticles. The same researchers found that three days after an intratracheal instillation 20 nm nickel particles caused a substantially higher increase in the LDH concentration (along with that of total protein and tumor necrosis factor-alpha) than 5 µm particles did [27].

As can be seen from the data presented in Table 2, copper-containing particles caused a similar effect, the increase in the LDH concentration being accompanied by an increase in the concentrations of three others lysosomal enzymes (amylase, alkaline phosphatase, and gamma-glutamyl transferase), which may be explained similarly.

**Table 2:** Some biochemical characteristics of the supernatant of the fluid obtained by bronchoalveolar lavage (BALF) of rats 24 h after intratracheal instillation of 0.5 mg of copper-containing NPs or submicron MPs in 1 mL of water suspension (*x* ± *S_x_*).

Indices	Groups of Rats Instilled Intratracheally with		
	Water (Control)	Nanoparticles	Submicron Particles
Glucose, mmol/L	0.56 ± 0.00	1.36 ± 0.25 *^,●^	0.56 ± 0.00
Urea nitrogen, mmol/L	0.36 ± 0.00	0.68 ± 0.10 *^,●^	0.36 ± 0.00
Uric acid, µmol/L	14.67 ± 0.33	16.75 ± 0.50 *^,●^	14.25 ± 0.25
Amylase, mcmol/L·min	30.00 ± 0.00	140.25 ± 3.50 *^,●^	30.00 ± 0.00
Alkaline phosphatase, mcmol/L·min	20.33 ± 2.60	29.75 ± 3.77	27.00 ± 1.68
ALT, mcmol/L·min	21.33 ± 0.67	25.25 ± 0.29 *^,●^	22.00 ± 0.41
AST, mcmol/L·min	7.33 ± 1.20	39.75 ± 2.29 *^,●^	19.50 ± 1.66 *
De Ritis ratio	0.34 ± 0.05	1.57 ± 0.08 *^,●^	0.88 ± 0.07 *
γ-Glutamyl transferase, mcmol/L·min	5.33 ± 0.33	14.50 ± 0.87 *	10.25 ± 0.63 *
Lactate dehydrogenase, mcmol/L·min	166.67 ± 30.33	821.50 ± 9.50 *^,●^	601.75 ± 54.65 *
Calcium, mmol/L	0.12 ± 0.00	0.25 ± 0.02 *^,●^	0.12 ± 0.00
Magnesium, mmol/L	0.12 ± 0.00	0.16 ± 0.01 *^,●^	0.12 ± 0.00
Phosphorus, mmol/L	0.14 ± 0.01	0.25 ± 0.02 *^,●^	0.14 ± 0.00
Iron, µmol/L	0.40 ± 0.00	0.83 ± 0.21 *^,●^	0.40 ± 0.00

Statistically significant difference * from control group; ^●^ from submicron particles group (*p* < 0.05 by Student’s *t*-test).

As follows from the same table, a statistically significant difference from the control values under the effect of NPs was also observed for a number of other indices, whereas under the effect of submicron MPs, corresponding shifts were either absent or less marked, inter-group differences being, as a rule, statistically significant. Indices that had no differences from the controls (total protein, albumin, creatinine, creatine kinase, and lipase) are not included in the Table 2.

Besides the cytotoxic action of NPs, another cause of all these shifts may be acute inflammatory changes in the pulmonary tissue induced by the copper, including by that released as ions, and related increase in vascular permeability. The latter mechanism is convincingly suggested by a rise in the concentration of all 4 measured cations, and the same mechanism of increase in BALF concentration is also most likely for glucose, urea and uric acid. At the same time, it is interesting that increased aminotransferases levels (ALT and, even to a greater degree, AST) were accompanied by an increase in their ratio (de Ritis coefficient). Such shifts cannot be well explained only by enhanced penetration of blood enzymes into BALF due to the increased vascular permeability; moreover, they correspond to the shifts in ALT and AST that are usually observed in the blood under a variety of chronic intoxications and are considered to be one of the markers of liver damage. It is difficult to assume, however, that such damage to the liver could have developed within only 24 h after intratracheal instillation of a low dose of NPs, all the more so that repeated i.p. administrations of the same NPs were not observed to cause any significant shifts in aminotransferase activities in blood serum. The ALT activity in controls was 52.80 ± 3.65 and in the exposed rats 49.25 ± 2.66, respective values for the AST activity were 254.70 ± 16.78 and 234.8 ± 13.29 mmol/h·L [17].

Further, the higher biological aggressivity of the copper-containing NPs tested by us in comparison with submicron MPs manifesting itself in response to a single intratracheal administration is demonstrated by the entire body of both cytological and biochemical data presented in Table 1 and Table 2**.**

### 2.3. Semi-Contact Atomic Force Microscopy (sc-AFM) and Transmission Electron Microscopy (TEM) Data

**Figure 2:**
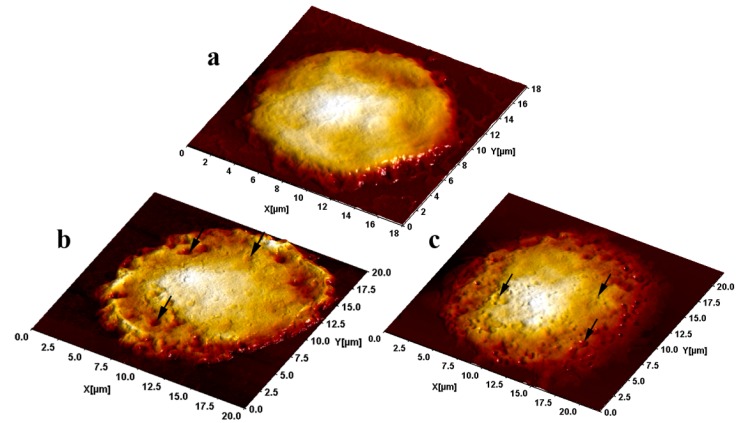
Alveolar macrophage surface topography visualized by the sc-AFM: (**a**) controls; (**b**) after instillation of 340 nm copper oxide-copper particles and (**c**) after instillation of 20 nm copper oxide particles. Typical pits are pinpointed by arrows.

Semi-contact AFM investigation of BALF cells from rats administered the particles under study revealed numerous pits on their surface compared with the virtually smooth surface of cells from control rats. The number of such pits was markedly larger after the instillation of NPs while the prevailing sizes of the pits were smaller than after that of submicron MPs. This visual assessment as illustrated by Figure 2 is confirmed by the statistics. Indeed, the mean pit diameter correlates with the mean diameter of phagocytized particles, being equal to (mean ± SE) 25.2 ± 0.9 nm in rats administered NPs and to 290 ± 14 nm in those administered MPs, the average number of pits per unit surface area (mcm^2^) being equal to 74.40 ± 5.30 and only 1.85 ± 0.50, respectively (for counts within small 2 × 2 µm scans ensuring the highest image resolution).

As is well known, the starting point for the engulfment of a particle by a phagocytic cell (endocytosis) is close contact between them, as a result of which a subjacent portion of the plasma membrane is as though indented (the so-called invagination) and then is pinched off forming a membrane-bound vesicle called an endosome or phagosome.

We proceeded from the assumption that the invagination process changes the topography of the phagocyte’s surface (be it of an alveolar macrophage or of a neutrophil leukocyte), leading to the formation of “a pit”. We demonstrated that this was indeed a fact first in experiments with nano- and microparticles of iron oxide Fe_3_O_4_ (magnetite) [17,18]. It was shown that both the quantity and the size of these pits depended on the predominant dimension of the particles being engulfed and on the phagocytic activity of the cell, which, in turn, depended on the particle’s cytotoxicity and thus, inversely, on its diameter again. This inverse dependence of phagocytic activity upon particle size was also demonstrated by assessing the internalized iron oxide particle load of AMs and NLs under optical microscopy [17,18].

Later on, we observed the same “pitting” phenomenon in a comparative experiment with nanosilver and nanogold suspensions [21]. In this case the NPs compared had virtually one and the same average diameter, and so the pit dimensions proved to be independent of the NPs’ chemical nature. However, the pit count per unit area of cell surface was much higher for more cytotoxic nanosilver than for less cytotoxic nanogold.

Assuming that each pit is a mark left by a single particle or by a small aggregate of particles that have just been engulfed by a cell, one may regard the average surface concentration of such marks as a comparative estimate of a cell’s phagocytic activity. Thus, the sc-AFM data suggest that NPs which, judging by the NL/AM index are more cytotoxic (due to a smaller size as in experiments with nanomagnetite, or to the chemical nature as in the case of nanosilver, or to both factors as in the present case of CuO) are engulfed more avidly. This phenomenon can be explained by the experimentally proven fact that macrophage breakdown products stimulate not only the recruitment but also the phagocytic activity of viable macrophages [25].

The additional importance of the sc-AFM data is that they indirectly confirm the presence of phagocytized CuO *nanoparticles* on the free surface of the lower airways immediately before the lavage was carried out 24 h after the pulmonary deposition of these particles. Meanwhile, as shown in Section 3, they were not to be discovered *in vitro* by the optical absorption method as soon as an hour and a half after the addition of the BALF supernatant to a highly stable water suspension of the same NPs (in a ratio of 1:3), which points to almost complete dissolution of these NPs. Thus, no such complete dissolution takes place *in vivo* even over a much longer time. This fact is of particular importance in the light of the above-mentioned debate as to whether nano-CuO cytotoxicity is associated with the action of particles as such or with that of Cu-ions released by their dissolution.

**Figure 3:**
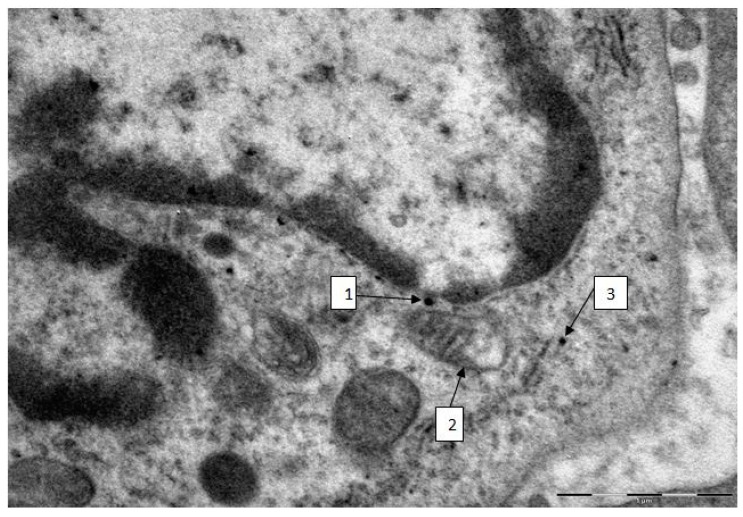
Alveolar macrophage from the BALF of a rat administered copper oxide NPs (TEM, magnification ×22,000). There is a solitary NP close to the nuclear membrane (arrow 1). Note that in the particle localisation area the membrane is blurred and lacks its double contour image. The mitochondrion near this particle (arrow 2) partly lacks cristae and the electron density of its matrix is reduced; the remaining cristae show signs of destruction, and the mitochondrial membrane is blurred, partly broken, and lacking double contour in some places. There is nearby a solitary nanoparticle within the “shadow” of a destroyed mitochondrion (arrow 3).

The presence of such persisting NPs *inside* AMs 24 h after instillation (added to which should be another 1.5 h lag between obtaining BALF and the fixation of the cell sediment with glutaraldehyde) is demonstrated expressly by the TEM data. However, the importance of these data is associated with estimating not only endocellular particles localization but also ultrastructural damage to phagocytizng cells caused by them. A typical damage to alveolar macrophages is illustrated by Figure 3, and we did not find any noticeable differences between macrophages and neutrophil leukocytes, in this respect. The TEM images of BALF cells obtained from control rats were normal.

Judging by the numerous data summarized in an extensive review [28], the targets for the cytotoxic action of various nanoparticles are the cell membrane (Ag, Au, CeO, SiO_2_, TiO_2_, iron oxide, quantum points), the nucleus (Ag, Au, CeO, SiO_2_, TiO_2_, ZnO), mitochondria (Ag, SiO_2_, ZnO), lysosomes (CeO, TiO_2_, ZnO), endoplasmic reticulum (Ag, SiO_2_), cytoskeleton (Ag, Au, CeO, SiO_2_, TiO_2_, quantum points), and, possibly, even the Golgi apparatus (TiO_2_, iron oxide). Usually, specific sites are suggested for the action of each type of NP. It should be stressed, however, that the above list of ultrastructural cell damage-inducing NPs is not exhaustive. For instance, our experiments with iron oxide [17,18], silver and gold [21] nanoparticles revealed in all the three cases damage to the nuclear membrane and particularly, to mitochondria.

Note that the review [28] does not mention copper containing NPs. Meanwhile, in our study transmission electronic microscopy of both AMs and NLs clearly revealed ultrastructural damage to the cell (in particular, to mitochondria, as well as to cellular and nuclear membranes) even where a very small number of particles were detected in it as illustrated by Figure 3.

## 3. Experimental Section

The animal experiments were carried out on outbred white female rats from our own breeding colony with the initial body weight of 150 to 220 g, with a minimum of 12 animals in different exposed and control groups. Rats were housed in conventional conditions, breathed unfiltered air, and were fed standard balanced food. The experiments were planned and implemented in accordance with the “International guiding principles for biomedical research involving animals” developed by the Council for International Organizations of Medical Sciences (1985) and approved by the Ethics Committee of the Ekaterinburg Medical Research Center Medical for Prophylaxis and Health Protection in Industrial Workers.

For this animal experiment, we prepared stable suspensions of copper oxide nanoparticles (NPs) and submicron microparticles (MPs).

NPs engineered by the method of laser ablation followed by concentration strengthening by partial evaporation proved necessary to enable the administration of effective doses to rats in minimal volumes of water. A plate of copper with a metal content of 99.99% was placed on the bottom of a dish with deionized water. Metal ablation was performed using an Fmark-20RL laser material processing system (by Laser Technology Center, Ekaterinburg, Russia), based on ytterbium-doped pulsed fiber laser (pulse length 100 ns, repetition rate 21 kHz, wavelength 1064 nm). The energy density was 80 J/cm^2^. The target was irradiated in scanning mode with a rate of the laser ray of 270 mm/s (the first 7 cycles of such scanning served to prepare the target’s surface).

**Figure 4:**
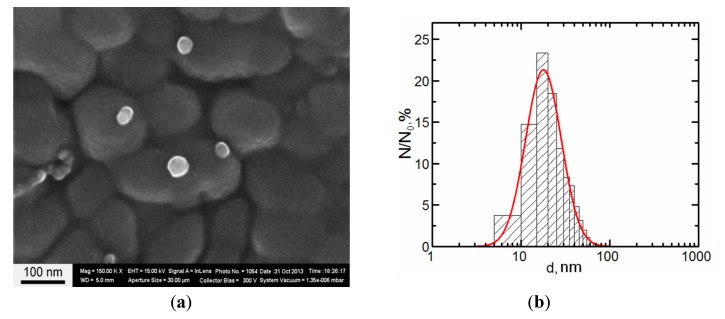
(**a**) Scanning electron microscopy (SEM) images of nanoparticles prepared for the experiment and (**b**) particle size distribution function obtained by analysis of SEM images (N—number of particles of a given diameter, N_0_—total number of particles).

The concentration of the primary suspensions obtained by ablation was 0.08 mg/mL. An increase in concentration to 0.5 mg/mL was achieved by drying the suspensions for 5 h at 50 °C, which was not accompanied by nanoparticle aggregation. Particle images were obtained after concentrating by scanning electron microscopy (SEM) with the AURIGA CrossBeam Workstation (Carl Zeiss, Jena, Germany), which enabled us to identify their spherical form (Figure 4). The average particle diameter (±SD) obtained through statistical processing of hundreds of SEM images was 20 ± 10 nm, the distribution being symmetrical.

No essential changes took place 30 days after the preparation of the suspension in either zeta potential or the form and position of the plasmon resonance peak, providing evidence of its high stability. For studying the kinetics of NP dissolution in an ionic medium or in a model liquid biological medium, we measured change in optical absorption at 620 nm wave length corresponding to maximum optical absorption of copper nanoparticles. Before measurement, the suspension of copper oxide nanoparticles with a concentration of 0.5 mg/mL was diluted fourfold by normal saline or by supernatant of a fluid obtained through bronchoalveolar lavage (BALF) of intact rats. Absorption spectra were measured by means of an UV-1650 spectrophotometer. Before each measurement, the solution was sonicated. We found that nanoparticles would completely vanish (presumably dissolving) within 20 min after the addition of normal saline and within 90 min after the addition of BALF.

The chemical composition of the nanoparticles was determined by X-ray energy dispersion analysis using an Auriga CrossBeam scanning electronic microscope (Carl Zeiss, Jena, Germany) equipped with an X-Max X-ray detector (Oxford Instruments, Oxford, UK). Analysis of the characteristic X-ray radiation resulting from the bombardment of a target surface with a beam of accelerated electrons makes it possible to determine the element composition in the area of interaction between electrons and the substance to a depth of several micrometers. Measurements were performed with an accelerating voltage of 5 kV and averaged over an area of 100 × 100 µm. The composition of the nanoparticles (in terms of the number of atoms) was found to be equal to 53% ± 5% of Cu and 47% ± 5% of O, *i.e.*, actually to 1:1, which corresponds to the chemical composition of nanoparticles in workroom air during the casting of refined copper (see Introduction) and is close to CuO. However, it should be noted that in both cases the NPs were unlikely to consist of CuO as a chemical compound, since it is characteristic of particles formed during vapor condensation, and generally of condensed phases, to display substantial deviations from stoichiometry. Most likely, these NPs consisted of a mix of various copper oxides, which are impossible to identify individually.

**Figure 5:**
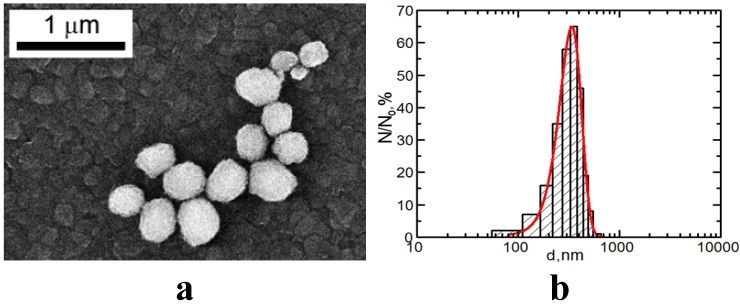
(**a**) Scanning electron microscopy (SEM) images of microparticles prepared for the experiment and (**b**) particle size distribution function obtained by analysis of SEM images (N—number of particles of a given diameter, N_0_—total number of particles).

Submicron MPs (Figure 5) were obtained by levigating under ultrasonication a water suspension of powder produced by electric explosion of a copper wire of the same 99.99% purity. The average particle diameter (±SD) obtained through statistical processing of hundreds of SEM images was 340 ± 168 nm.

Using X-ray energy dispersion analysis we found that the 80 nm surface layer of a submicron MP had a Cu:O atomic ratio corresponding to copper(I) oxide (Cu_2_O), while the nucleus of the particle was the element copper (Cu). In suspension on de-ionzied water, MP keeps this structure for a long time. Meantime, in a suspension with the supernatant of BALF from intact rats added to it (in a ratio of 1:3) it loses the copper oxide layer within 24 h through dissolution, which results in a reduced mean particle diameter to 175 nm, while the remaining metallic nucleus practically does not dissolve.

Bronchoalveolar lavage (BAL) was carried out 24 h after the i.t. instillations of the NP or MP suspension or reference water. A cannula connected to a Lüer’s syringe containing 10 mL of normal saline was inserted into the surgically prepared trachea of a rat under hexenal anesthesia. The BAL fluid (BALF) entered the lungs slowly under the gravity of the piston, with the animal and syringe positioned vertically. Then the rat and the syringe were turned 180°, and the fluid flowed back into the syringe. The extracted BALF was poured into siliconized refrigerated tubes. An aliquot sample of the BALF was drawn into a WBC count pipette together with 3% acetic acid and methylene blue. Cell count was performed in a standard hemocytometer (the so-called Goryayev’s Chamber). Along with optical and transmission electron microscopy of cells sedimented by centrifuging the BALF, we examined the topography of the BALF cell surfaces with the help of semi-contact atomic force microscopy (sc-AFM) reputed as a unique technique allowing one to obtain 3D visualizations of the surface topography of biological objects with a nanometric spatial resolution. Transmission electron microscopy (TEM) was used to study the localization of different NPs within the BALF phagocytes and to visualize damage to the cells at ultra structural level that may be attributed to the cytotoxic effect of NPs.

For optical microscopy, the BALF was centrifuged for 4 min at 1000 rpm before the fluid was decanted, and the sediment was used for preparing smears on 2 microscope slides. After air-drying, the smears were fixed with methyl alcohol and stained with azure eosin. The differential count (under an optical microscope with immersion at a magnification of 1000×) for determining the percentage of alveolar macrophages (AM), neutrophil leukocytes (NL) and other cells was conducted up to a total number of 100 counted cells. Allowing for the total number of cells in the BALF, these percentages were recalculated in terms of absolute AM and NL counts. The supernatant of the centrifuged BALF was used for biochemical testing, the results of which are given in Table 2.

For performing TEM, BALF was centrifuged for 30 min at 3000 rpm. The cell sediment was fixed in 2.5% solution of glutaraldehyde with subsequent additional fixing in 1% solution of osmium tetroxide for 2 h. Then it was washed in 0.2 M phosphate buffer and passed through alcohols of increasing concentration and through acetone for dehydration. Then the sample was placed for 24 h in a mixture of araldite and acetone at a ratio of 1:1, following which it was polymerized in araldite at 37 °C for 1 day and at 50–60 °C for the next 2–3 days. Ultrathin sections were obtained on a Leica EM UC6 ultra microtome (Leica Microsystems GmbH, Wetzlar, Germany), contrasted with lead citrate and examined on a Morgagni 268 electron microscope (FEI Company, Eindhoven, The Netherlands).

For sc-AFM, the BALF was centrifuged for 4 min at 1000 rpm, and a 3 μL aliquot of the BALF sediment was precipitated on a fresh cleavage of mica. After 60 s, the excessive suspension was removed with a paper filter, and the sample was dried by blowing with clean, dry air or nitrogen for 30 s. It should be noted that the drying of the BALF on a mica surface results in the formation of salt microcrystals, which were removed by washing the sample twice. For washing, the sample was kept for 60 s on the surface of a drop of deionized water (with the working side down). The liquid was then removed with the help of a paper filter. After repeating the washing, the sample was dried by blowing with clean, dry air or nitrogen for 30 s. Investigation of the cell surface morphology was performed by sc-AFM with the help of an NTEGRA Therma scanning probe platform produced by the NT-MDT Company (Zelenograd, Russia) using semi-contact atomic force microscopy mode with NSG01 probes by the same producer. The height of the probes was about 15 μm, and the tip curvature radius was less than 10 nm. For statistical processing and analysis of measurement results, we used specialized software, SPIP (Image Metrology, Horsholm, Denmark) and SIAMS Photolab (SIAMS Company, Ekaterinburg, Russia). The procedures developed made it possible to reveal the pits in the images of cell surfaces and to measure the diameter of each pit. The results of the image analysis were used for plotting the histograms of the pit dimensions for the cells of all groups of rats. The sc-AFM gives no reliable distinction between AMs and NLs. Since taking all cells of lesser dimensions as neutrophils would be too arbitrary, we took as alveolar macrophages only the largest BALF cells (and present in this paper only pits measured and counted on their surface).

## 4. Conclusions

The presence of ultrafine (submicron) particles having sizes both within the conventional nano-range and over its upper boundary (100 nm) in the aerosols polluting the workplace air at high-temperature metallurgical technologies (in particular, copper smelting and casting) makes it important to compare the health impact of such particles. The findings of our study based on the intratracheal instillation of particles in sterile water suspensions provide evidence that both fractions of copper-containing ones, but of nanoparticles especially, evoke an active recruitment of alveolar macrophages (AM) and, to a greater extent, of neutrophil leukocytes (NL). These cells feature a high phagocytic activity in relation to submicron particles and, to a much greater extent, to nanoparticles. At the same time, the latter cause a substantially higher increase in the NL/AM ratio, along with biochemical shifts in the bronhoalveolar lavage fluid, testifying to their higher cytotoxicity and, possibly, higher toxicity for the pulmonary tissue in general (pulmonary toxicity).

We believe that the cytotoxic effect is likely to be only partly associated with particle dissolution which, in the case of nanoparticles, is more intensive, since electron microscopy reveals explicit association between the ultrastructural damage to the cell and intracellular localization of persisting particles. In particular, the pronounced damage to the mitochondria is in agreement with the widespread concept stressing the role of oxidative burst as an important mechanism of the cytotoxic action of metallic, including copper-containing, nanoparticles.
